# Behavioral and clinical correlates of serum bilirubin concentrations in Japanese men and women

**DOI:** 10.1186/1472-6823-13-39

**Published:** 2013-10-04

**Authors:** Maya Tanaka, Sanjeev Budhathoki, Akie Hirata, Makiko Morita, Suminori Kono, Masahiro Adachi, Hisaya Kawate, Keizo Ohnaka, Ryoichi Takayanagi

**Affiliations:** 1 Department of Preventive Medicine, Graduate School of Medical Sciences, Kyushu University, Maidashi 3-1-1, Higashi-ku, Fukuoka, 812-8582, Japan; 2 Department of Medicine and Bioregulatory Science, Graduate School of Medical Sciences, Kyushu University, Fukuoka, Japan; 3 Department of Geriatric Medicine, Graduate School of Medical Sciences, Kyushu University, Fukuoka, Japan

**Keywords:** Bilirubin, Coffee, Smoking, Alcohol, High-density lipoprotein cholesterol

## Abstract

**Background:**

A considerable interest has been drawn to potential protective effects of bilirubin against oxidative stress-related diseases. Smoking is known to be associated with lower concentrations of serum bilirubin, but other behavioral correlates of serum bilirubin have not been well studied. In this cross-sectional study, we examined the associations of behavioral and clinical factors with serum total bilirubin in Japanese men and women.

**Method:**

The study subjects comprised of 4802 men and 6414 women aged 49–76 years who participated in the baseline survey of an ongoing cohort study on lifestyle-related diseases in Fukuoka, Japan. With consideration to time of the day of blood sampling and fasting hours, the associations with smoking, alcohol intake, body mass index, physical activity, coffee, tea, blood pressure, glycated hemoglobin (HbA1c), HDL cholesterol and non-HDL cholesterol with serum bilirubin were evaluated by analysis of covariance and multiple linear regression analysis.

**Results:**

While smoking was negatively associated with serum bilirubin, alcohol consumption was positively associated with serum bilirubin in both men and women. Coffee consumption was associated with lower bilirubin concentrations in both sexes. In the multiple linear regression analysis, HDL cholesterol was positively and HbA1c was negatively associated with bilirubin in both men and women, and the associations were more evident in women.

**Conclusion:**

Smoking, alcohol use and coffee consumption were important behavioral correlates of serum bilirubin in Japanese men and women. Serum HDL cholesterol was a measurable clinical correlate of bilirubin in women.

## Background

A considerable interest has been drawn to potential protective effects of bilirubin against oxidative stress-related diseases [1-4]. High bilirubin concentrations have been related to decreased risk of coronary artery disease [1-4] while evidence remains controversial as regards the associations with stroke and cancer risk [2,4]. High bilirubin concentrations may also be protective against the deterioration of glucose metabolism [5]. Serum bilirubin concentrations are low in women than in men [6-8], and seem to decrease with age in adulthood at least in men [6,7]. Smoking is known to be associated with lower concentrations of serum bilirubin [6-9], but other behavioral correlates of serum bilirubin have not been well studied.

In epidemiological studies on bilirubin and disease risk, the investigators examined the associations of behavioral and clinical factors with serum bilirubin mostly in the univariate analysis [6,7,10-12] or in the analysis controlling for demographic factors only [12,13] in order to explore potential confounders. Only two studies performed the multivariate analysis on serum bilirubin in relation to cardiovascular risk factors [14,15]. A negative association between body mass index (BMI) and serum bilirubin was observed in several [7,10-13], but not all [6,14,15], of these studies. Few studies suggested that alcohol use was related to higher concentrations of serum bilirubin [7,12], but such an association was not found in other studies [11,14]. It was reported that high-density lipoprotein (HDL) cholesterol was positively correlated with serum bilirubin while total or non-HDL cholesterol was negatively correlated with bilirubin [11-14,16,17], but the findings are not consistent [10,15].

In this cross-sectional study, we examined the associations of behavioral and clinical factors with serum bilirubin in Japanese men and women. Behavioral factors included coffee and tea consumption in addition to smoking, alcohol use, BMI, and physical activity. Coffee and green tea are abundant in antioxidant polyphenols, and the consumption of these beverages has been implicated as protective against various diseases [18,19].

## Methods

### Study subjects

The study subjects comprised of men and women who participated in the baseline survey of an on-going cohort study on lifestyle-related diseases. Residents of the East Ward of Fukuoka City aged 50 to 74 years were invited to participate in the study by mail. All participants were informed of the details of the survey and gave written informed consent prior to their participation in the study. The study was approved by the Ethics Committee of the Kyushu University Faculty of Medical Sciences.

During the period from February 2004 to August 2007, a total of 12948 subjects participated in the baseline survey, with a participation rate of 24%. Age of the participants at the time of survey ranged from 49 to 76 years. Stored serum samples of 12942 participants were available for the measurement of bilirubin. We excluded 1548 participants who were under medical care for coronary artery disease (*n* = 533), stroke (*n* = 251), arteriosclerosis obliterance (*n* = 36), chronic liver disease (*n* = 252), cancer (*n* = 496), alcohol abuse (*n* = 4) or who had aspartate aminotransferase (AST) or alanine aminotransferase (ALT) greater than 3-fold of the upper limit of the normal range, i.e., > 120 U/L (*n* = 59), total bilirubin > 3 mg/dL (*n* = 3), creatinine > 2 mg/dL (*n* = 44). Additionally excluded were 160 subjects who reported a prior history of coronary artery disease (*n* = 31) or stroke (*n* = 131) without current medical care. We further excluded 18 participants with missing values on the covariates under consideration. The remaining 11216 subjects (4802 men and 6414 women) were included in the present analysis.

### Baseline survey

At the baseline survey, each participant completed a self-administered questionnaire and underwent measurements of blood pressure, height (cm) and body weight (kg). Fasting or nonfasting venous blood was drawn. The questionnaire inquired about smoking habits, alcohol consumption, physical activity, dietary intake, disease history, and use of selected drugs and supplements. Details of the questionnaire have been described previously [20,21]. In brief, ever-smoking was defined as having smoked at least one cigarette per day for one or more years. Past and current smokers reported the number of cigarettes smoked per day and years of smoking. Ever-alcohol use was defined as having drunk alcoholic beverages at least once a week for one or more years. Past alcohol use was separated from lifelong alcohol-abstinence. Current alcohol drinkers reported consumption frequencies and amounts of 5 alcoholic beverages on average in the past year, and the amount of ethanol consumed per day was calculated. Questions on physical activity ascertained work-related (4 types) and leisure-time (3 types) physical activities over the previous year. With consideration to intensity in terms of metabolic equivalent (MET) and amount of time for each physical activity, MET-hours were calculated for work-related and leisure-time physical activity separately. BMI was calculated as weight (kg) divided by squared height (m^2^). The time of the last meal prior to the survey and the time of blood drawing were recorded.

Systolic and diastolic blood pressure were measured by an automated digital device (HEM-707, OMRON, Kyoto) after at least five minutes of sitting position. Blood pressure was taken twice with an interval of at least one-minute, and the second reading was used in this study.

### Laboratory measurements

Serum samples stored at −80°C were used for the determination of total, unconjugated and conjugated bilirubin concentrations. Measurements of bilirubin were performed by the using absorptiometric assay with chemical oxidation by vanadic acid [22] at an external laboratory (SRL, Hachiohji, Japan). Total bilirubin was used in the present study. HbA1c was measured at the above-described laboratory by using the latex agglutination immunoassay [23]. As for eight items of serum biochemistry including AST, ALT, total and HDL cholesterol and creatinine, recorded information was referred to if these measurements had been done in the past year. When recorded information was not available, 5 mL of venous blood was taken for the measurements, and serum samples frozen in dry ice were shipped to the above-mentioned external laboratory.

### Statistical analyses

**Figure 1:**
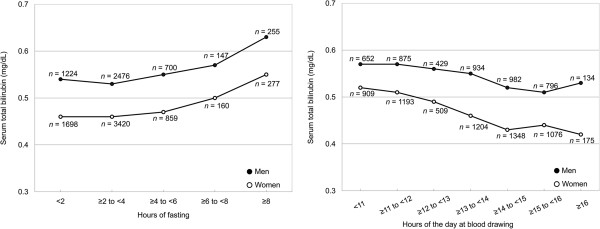
**Geometric means of serum total bilirubin according to hours of fasting (left panel) and time of blood drawing (right panel) in men (*n *= 4802) and women (*n *= 6414).** Adjusted for age and either time of blood drawing or hours of fasting. *P* < 0.0001 for both men and women in both models.

The distribution of serum total bilirubin was skewed to the right side, and the values were always transformed to the natural logarithms in the analysis. Fasting status [24,25] and hours of the day [26] are known to affect bilirubin concentrations. Thus we did a preliminary analysis on fasting hours and the time of blood drawing in relation to bilirubin concentrations to determine the category of these factors in the main analysis. Based on the patterns shown in Figure 1, the fasting hours were collapsed into < 4, 4–7 and ≥ 8 hours, and the time of blood drawing was classified into < 12, 12–14 and ≥ 14 hours. Bilirubin concentrations also differed slightly by season as reported previously [27]. With adjustment for age, fasting hours and time of blood drawing, geometric means for winter (December to February), spring (March to May), summer (June to August) and autumn (September to November) were 0.53, 0.53, 0.56 and 0.55 mg/dL respectively among men (analysis of covariance *P* = 0.0005), and 0.46, 0.46, 0.48, and 0.48 mg/dL respectively among women (*P* = 0.01). Seasons were thus collapsed into 2 categories (winter/spring and summer/autumn).

As clinical covariates, hypertension, type 2 diabetes mellitus and elevated non-HDL cholesterol were defined. Hypertension was defined if systolic blood pressure was ≥ 140 mmHg or diastolic blood pressure was ≥ 90 mmHg or if antihypertensive drugs were used. Type 2 diabetes mellitus was defined if individuals reported current medication for diabetes mellitus or if HbA1c was ≥ 6.5% [28]. Elevated non-HDL cholesterol was defined if non-HDL cholesterol was ≥ 170 mg/dL [29].

Analysis of covariance was used to calculate adjusted geometric means and 95% confidence intervals (CI) of total bilirubin concentrations according to smoking (never, past and current smokers with < 15, 15–29 and ≥ 30 cigarettes/day), alcohol intake (never, past and current drinkers with a consumption of < 25, 25–49 and ≥ 50 g of alcohol/day), BMI (< 22.5, 22.5–24.9, 25.0–27.4 and ≥ 27.5 kg/m^2^), job-related and leisure-time physical activity (sex-specific quartiles each), coffee (0, < 1, 1–3, 4–6 and ≥ 7 cups/day), tea (< 1, 1–3, 4–6, 7–9 and ≥ 10 cups/day), hypertension, type 2 diabetes mellitus and elevated non-HDL. Three models were used in the statistical adjustment. The first model included only age (continuous variable) as covariate; the second model used age, hours of fasting (< 4, 4–7 and ≥ 8 hours), time of blood drawing (< 12, 12–14 and ≥ 14 hours), and season (winter/spring and summer/autumn) as covariates; and the third model included all of the above-mentioned lifestyle and clinical covariates as well as those used in the model 2. In the analysis of covariance, indicator variables were used for each categorical factor except for a factor of interest. The Dunnett method was used for the two-group comparison with the lowest category as referent.

Multiple linear regression analysis was employed in the analysis using continuous variables for diastolic blood pressure, non-HDL cholesterol, HDL cholesterol and HbA1c and also including drug uses as covariates. Categorical covariates were represented by indicator variables. The relative contribution of a specific covariate to the variation in bilirubin concentrations was expressed by the proportion of the partial *R*^*2*^ to the overall *R*^*2*^ estimated in the model including all of the covariates. The partial *R*^*2*^ was estimated as the difference in the *R*^*2*^ statistics between the full model and a model including all the covariates except the specified variable. Because the number of the subjects was fairly large, two-tailed P value < 0.01 was considered as statistically significant. All statistical analyses were carried out by using SAS version 9.2 (SAS Institute, Cary, NC).

## Results

Mean ages were 62.5 years (SD 6.8 years) in men and 62.0 years (SD 6.7 years) in women. Geometric mean concentrations of serum bilirubin were 0.54 mg/dL in men and 0.47 mg/dL in women.

With respect to each of the lifestyle and clinical factors under study, the geometric means of serum bilirubin adjusted for age, postprandial hours, time of blood drawing and season (model 2) were almost the same as the age-adjusted geometric means (model 1). Thus age-adjusted and multivariate-adjusted geometric means (model 3) are shown for ease of presentation.

In men (Table 1), serum bilirubin was progressively lower with increasing amounts of cigarettes per day while alcohol consumption was strongly positively associated with serum bilirubin. In women (Table 2), serum bilirubin was also lower in current smokers, but there was no dose-relationship with the amount of cigarettes. Women also showed a positive association between alcohol consumption and bilirubin. Past alcohol drinkers had rather lowered concentrations of bilirubin in women. BMI was unrelated to serum bilirubin in men, but women showed statistically, significantly lower concentrations in the highest BMI category (≥ 27.5 kg/m^2^) as compared with the lowest category.

**Table 1:** Serum total bilirubin (mg/dL) according to behavioral and clinical factors in men

**Variable**	**Category**	**n**	**Geometric mean (95% CI)**	
			**Age-adjusted**	**Multivariate-adjusted**^**a**^
Smoking (cigarettes/day)	Never	1245	0.58 (0.57−0.59)	0.58 (0.57−0.59)
	Past	2038	0.57 (0.56−0.58)	0.57 (0.56−0.58)
	< 15	262	0.51 (0.49−0.54)^h^	0.51 (0.49−0.53)^h^
	15–29	873	0.48 (0.47−0.49)^h^	0.48 (0.47−0.49)^h^
	≥ 30	384	0.47 (0.45−0.49)^h^	0.47 (0.45−0.49)^h^
	*P*-value^b^		<0.0001	<0.0001
Alcohol intake (g/day)	Never	1020	0.50 (0.49−0.51)	0.50 (0.49−0.52)
	Past	301	0.49 (0.47−0.51)	0.49 (0.47−0.52)
	< 25	1472	0.55 (0.54−0.56)^h^	0.54 (0.53−0.55)^h^
	25–49	1229	0.57 (0.55−0.58)^h^	0.56 (0.55−0.58)^h^
	≥ 50	780	0.58 (0.57−0.60)^h^	0.59 (0.57−0.60)^h^
	*P*-value^b^		<0.0001	<0.0001
Body mass index (kg/m^2^)	< 22.5	1663	0.54 (0.53−0.55)	0.54 (0.53−0.55)
	22.5–24.9	1780	0.54 (0.53−0.55)	0.54 (0.53−0.55)
	25.0–27.4	1004	0.56 (0.54−0.57)	0.55 (0.54−0.56)
	≥ 27.5	355	0.54 (0.52−0.56)	0.54 (0.52−0.56)
	*P*-value^b^		0.14	0.57
Job-related physical activity^c^	Q1	1471	0.54 (0.53−0.55)	0.55 (0.54−0.56)
	Q2	1104	0.55 (0.53−0.56)	0.55 (0.53−0.56)
	Q3	1087	0.55 (0.53−0.56)	0.54 (0.53−0.55)
	Q4	1140	0.53 (0.52−0.55)	0.53 (0.52−0.54)
	*P*-value^b^		0.40	0.23
Leisure-time physical activity^c^	Q1	1112	0.52 (0.51−0.54)	0.54 (0.52−0.55)
	Q2	1312	0.54 (0.53−0.55)	0.54 (0.53−0.55)
	Q3	1178	0.55 (0.53−0.56)	0.54 (0.53−0.55)
	Q4	1200	0.56 (0.55−0.57)^h^	0.55 (0.54−0.56)
	*P*-value^b^		0.0007	0.34
Coffee (cups/d)	0	814	0.58 (0.57−0.60)	0.57 (0.55−0.58)
	< 1	1250	0.57 (0.56−0.58)	0.56 (0.55−0.57)
	1–3	2149	0.53 (0.53−0.54)^h^	0.54 (0.53−0.55)^g^
	4–6	497	0.48 (0.46−0.50)^h^	0.51 (0.49−0.52)^h^
	≥ 7	92	0.44 (0.41−0.48)^h^	0.48 (0.45−0.52)^h^
	*P*-value^b^		< 0.0001	< 0.0001
Tea (cups/d)	< 1	638	0.54 (0.52−0.56)	0.54 (0.52−0.56)
	1–3	1969	0.55 (0.54−0.56)	0.55 (0.54−0.56)
	4–6	1379	0.55 (0.53−0.56)	0.54 (0.53−0.56)
	7–9	483	0.52 (0.50−0.54)	0.52 (0.51−0.54)
	≥10	333	0.55 (0.52−0.57)	0.55 (0.53−0.57)
	*P*-value^b^		0.18	0.23
Hypertension^d^	(−)	1859	0.54 (0.53−0.55)	0.55 (0.54−0.56)
	(+)	2943	0.54 (0.54−0.55)	0.54 (0.53−0.55)
	*P*-value^b^		0.34	0.20
Type 2 DM^e^	(−)	4321	0.55 (0.54−0.55)	0.54 (0.54−0.55)
	(+)	481	0.52 (0.50−0.53)	0.52 (0.51−0.54)
	*P*-value^b^		0.004	0.03
Elevated non-HDL cholesterol^f^	(−)	3554	0.55 (0.55−0.56)	0.55 (0.54−0.56)
	(+)	1248	0.51 (0.50−0.53)	0.52 (0.51−0.53)
	*P*-value^b^		< 0.0001	< 0.0001

CI, confidence interval; DM, diabetes mellitus; HDL, high-density lipoprotein.

^a^Adjusted for age (year), hours of fasting (< 4, 4–7 and ≥ 8 hours), time of blood drawing (< 12, 12–14 and ≥ 14 hours), season (winter/spring and summer/autumn) and the listed factors by analysis of covariance.

^b^Overall difference.

^c^Quartile category of MET-hours per day (job-related physical activity) or per week (leisure-time physical activity).

^d^Systolic blood pressure ≥ 140 mmHg or diastolic blood pressure ≥ 90 mmHg or use of antihypertensive drugs.

^e^HbA1c ≥ 6.5% or medication for diabetes mellitus.

^f^Non-HDL cholesterol ≥ 170 mg/dL.

^g^*P* < 0.01 and ^h^*P* < 0.001 as compared with the lowest category (Dunnett method).

**Table 2:** Serum total bilirubin (mg/dL) according to behavioral and clinical factors in women

**Variables**	**Category**	**N**	**Geometric mean (95% CI)**	
			**Age-adjusted**	**Multivariate-adjusted**^**a**^
Smoking (cigarettes/day)	Never	5685	0.47 (0.47−0.48)	0.47 (0.47−0.48)
	Past	331	0.46 (0.44−0.47)	0.46 (0.44−0.48)
	< 15	178	0.42 (0.39−0.44)^h^	0.41 (0.39−0.44)^h^
	15–29	195	0.40 (0.38−0.42)^h^	0.40 (0.38−0.42)^h^
	≥ 30	25	0.44 (0.38−0.51)	0.43 (0.37−0.50)
	*P*-value^b^		< 0.0001	< 0.0001
Alcohol intake (g/day)	Never	4523	0.47 (0.46−0.47)	0.46 (0.46−0.47)
	Past	158	0.43 (0.41−0.46)	0.44 (0.42−0.47)
	< 25	1499	0.48 (0.47−0.48)	0.48 (0.47−0.49)
	25–49	189	0.51 (0.48−0.53)	0.52 (0.50−0.55)^h^
	≥ 50	45	0.52 (0.47−0.58)	0.56 (0.50−0.62)^g^
	*P*-value^b^		0.0001	< 0.0001
Body mass index (kg/m^2^)	< 22.5	3361	0.47 (0.47−0.48)	0.47 (0.47−0.48)
	22.5–24.9	1810	0.47 (0.46−0.48)	0.47 (0.46−0.48)
	25.0–27.4	803	0.46 (0.45−0.47)	0.46 (0.45−0.48)
	≥ 27.5	440	0.43 (0.42−0.45)^h^	0.44 (0.42−0.45)^h^
	*P*-value^b^		< 0.0001	< 0.0001
Job-related physical activity^c^	Q1	1396	0.46 (0.45−0.47)	0.46 (0.46−0.47)
	Q2	2011	0.47 (0.46−0.48)	0.47 (0.46−0.47)
	Q3	1373	0.47 (0.46−0.48)	0.46 (0.46−0.47)
	Q4	1634	0.48 (0.47−0.48)	0.48 (0.47−0.49)
	*P*-value^b^		0.16	0.12
Leisure-time physical activity^c^	Q1	1684	0.45 (0.45−0.46)	0.46 (0.45−0.47)
	Q2	1720	0.48 (0.47−0.49)^h^	0.48 (0.47−0.48)^g^
	Q3	1375	0.47 (0.46−0.48)	0.47 (0.46−0.48)
	Q4	1635	0.47 (0.47−0.48)^g^	0.47 (0.46−0.48)
	*P*-value^b^		0.0006	0.02
Coffee (cups/d)	0	982	0.49 (0.48−0.51)	0.49 (0.48−0.50)
	< 1	1725	0.48 (0.47−0.49)	0.48 (0.47−0.49)
	1–3	3159	0.46 (0.46−0.47)^h^	0.46 (0.46−0.47)^h^
	4–6	484	0.43 (0.42−0.44)^h^	0.44 (0.42−0.45)^h^
	≥ 7	64	0.46 (0.42−0.50)	0.46 (0.42−0.50)
	*P*-value^b^		< 0.0001	< 0.0001
Tea (cups/d)	< 1	440	0.46 (0.44−0.47)	0.47 (0.45−0.48)
	1–3	1856	0.47 (0.46−0.48)	0.47 (0.46−0.48)
	4–6	2202	0.47 (0.46−0.48)	0.47 (0.46−0.48)
	7–9	1162	0.47 (0.46−0.48)	0.47 (0.46−0.48)
	≥ 10	754	0.48 (0.46−0.49)	0.47 (0.46−0.48)
	*P*-value^b^		0.52	0.95
Hypertension^d^	(−)	3465	0.47 (0.46−0.47)	0.47 (0.46−0.47)
	(+)	2949	0.47 (0.47−0.48)	0.47 (0.47−0.48)
	*P*-value^b^		0.25	0.29
Type 2 DM^e^	(−)	6151	0.47 (0.47−0.47)	0.47 (0.47−0.47)
	(+)	263	0.45 (0.43−0.47)	0.45 (0.43−0.47)
	*P*-value^b^		0.08	0.14
Elevated non-HDL^f^	(−)	4203	0.47 (0.46−0.47)	0.47 (0.46−0.47)
	(+)	2211	0.47 (0.46−0.47)	0.47 (0.46−0.48)
	*P*-value^b^		0.66	0.98

CI, confidence interval; DM, diabetes mellitus; HDL, high-density lipoprotein.

^a^Adjusted for age (year), hours of fasting (< 4, 4–7 and ≥ 8 hours), time of blood drawing (< 12, 12–14 and ≥ 14 hours), season (winter/spring and summer/autumn) and the listed factors by analysis of covariance.

^b^Overall difference.

^c^Quartile category of MET-hours per day (job-related physical activity) or per week (leisure-time physical activity).

^d^Systolic blood pressure ≥ 140 mmHg or diastolic blood pressure ≥ 90 mmHg or use of antihypertensive drugs.

^e^HbA1c ≥ 6.5% or medication for diabetes mellitus.

^f^Non-HDL cholesterol ≥ 170 mg/dL.

^g^*P* < 0.01 and ^h^*P* < 0.001 as compared with the lowest category (Dunnett method).

Age-adjusted, but not multivariate-adjusted, geometric means of serum bilirubin were higher in men with higher levels of leisure-time physical activity. Neither age-adjusted nor multivariate-adjusted means showed such a measurable association in women. On the other hand, job-related physical activity did not show any association with bilirubin concentrations in either men or women. In both men and women, with an exception of women consuming ≥7 cups of coffee, serum bilirubin was markedly lower with increasing consumption of coffee. The negative association between coffee and bilirubin was more evident in men than in women. The consumption of tea was unrelated to serum bilirubin in both sexes. In men, but not in women, type 2 diabetes mellitus and elevated non-HDL cholesterol were associated with lower bilirubin concentrations. Hypertension was not associated with serum bilirubin in either men or women.

We evaluated whether deterioration of liver function accounted for the positive association between alcohol and serum bilirubin. Medians of serum AST were 22 U/L (interquartile range [IQR] 19–27 U/L) in men and 21 U/L (19–25) in women, and those of serum ALT were 21 U/L (16–28 U/L) in men and 17 U/L (14–22 U/L) in women. Alcohol consumption was positively correlated with AST in men, and more weakly in women. Spearman correlation coefficients with alcohol consumption (excluding past drinkers) were 0.18 for AST and 0.01 for ALT in men, and 0.04 for AST and 0.002 for ALT in women. In men, with adjustment for the covariates except alcohol use in Table 1, geometric means of serum bilirubin for the first to fourth quartile categories of AST were 0.52, 0.54, 0.55 and 0.56 mg/dL (*P* < 0.0001), and those for the ALT categories were 0.53, 0.54, 0.54 and 0.56 (*P* = 0.02). Neither AST nor ALT was associated with bilirubin in women. With additional adjustment for AST (quartile categories), the multivariate-adjusted geometric means of serum bilirubin according to alcohol consumption (never, past, <25, 25–49 and ≥ 50 g/day) were 0.51, 0.50, 0.54. 0.56 and 0.58 U/L (*P* < 0.0001) in men and exactly the same as shown in Table 2 in women (*P* < 0.0001). The adjustment for ALT did not change the association between alcohol and bilirubin in either men or women (data not shown).

We performed a multiple linear regression analysis using continuous variables for diastolic blood pressure, non-HDL cholesterol, HDL cholesterol and HbA1c and also including drug uses as covariates (Table 3). Non-HDL cholesterol and use of cholesterol-lowering drug were negatively associated with serum bilirubin in men, but not in women. On the other hand, HDL cholesterol was strongly, positively associated with bilirubin in both men and women. Both genders showed a negative association between HbA1c and bilirubin. The associations with HDL cholesterol and HbA1c were more evident in women.

**Table 3:** Multiple linear regression of serum total bilirubin (mg/dL) in natural logarithm on clinical covariates

**Covariate**	**Men**		**Women**	
	**β (SE)**	***P***	**β (SE)**	***P***
Diastolic BP (per 10 mmHg)	0.0061 (0.0049)	0.22	0.0100 (0.0040)	0.01
Non-HDL cholesterol (per 10 mg/dL)	−0.0078 (0.0017)	< 0.0001	0.0018 (0.0014)	0.19
HDL cholesterol (per 10 mg/dL)	0.0165 (0.0040)	< 0.0001	0.0252 (0.0029)	< 0.0001
HbA1c (per 1.0%)	−0.0187 (0.0069)	0.007	−0.0411 (0.0087)	< 0.0001
Use of antihypertensives	−0.0313 (0.0138)	0.02	0.0051 (0.0124)	0.68
Use of cholesterol-lowering drug	−0.0779 (0.0201)	0.0001	0.0258 (0.0128)	0.05
Use of antidiabetics	−0.0070 (0.0256)	0.78	0.0467 (0.0309)	0.13

BP, blood pressoure; HDL, high-density lipoprotein.

Included in the model were age (year), hours of fasting (< 4, 4–7 and ≥ 8 hours), time of blood drawing (< 12, 12–14 and ≥ 14 hours), season (winter/spring and summer/autumn), smoking (never, past and current smokers with < 15, 15–29 and ≥ 30 cigarettes/day), alcohol intake (never, past and current drinkers with a consumption of < 25, 25–49 and ≥ 50 g/day), body mass index (< 22.5, 22.5–24.9, 25.0–27.4 and ≥ 27.5 kg/m ^2^), job-related and leisure-time physical activities (quartiles), coffee (0, < 1, 1–3, 4–6 and ≥ 7 cups/day), tea (< 1, 1–3, 4–6, 7–9 and ≥ 10 cups/day) and all of the listed variables.

The covariates in the multiple linear regression accounted for 12.9% of the variation of bilirubin concentrations in men and for 10.3% of that in women. The contribution of time of the day at blood sampling and fasting hours was relatively large, especially in women (Table 4). Comparatively, the seasonal variation that serum bilirubin was lower in winter/spring made a much smaller contribution. Smoking showed the largest contribution to the variation of bilirubin in men, and the proportion ascribed to smoking was much smaller in women. Alcohol use and coffee consumption were modest, but discernible contributors in men and women. The contribution of the other behavioral factors was negligible or at most minimal. Of the clinical correlates, HDL cholesterol was the most important covariate in women. The effect of non-HDL cholesterol and use of cholesterol-lowering was almost negligible in women. In men, non-HDL cholesterol, HDL cholesterol and use of cholesterol-lowering drug each showed a small contribution to almost the same extent. The contribution of HbA1c was less important as determinant of bilirubin concentrations in men.

**Table 4:** Relative contribution of behavioral and clinical correlates to the variation of total bilirubin concentrations

**Variable (category or unit)**	**Contribution fraction (%)**^**a**^	
	**Men**	**Women**
Fasting hour (< 4, 4–7 and ≥ 8 hours)	7.3	10.2
Time of blood drawing (< 12, 12–14 and ≥ 14 hours)	10.6	33.2
Season (winter/spring and summer/autumn)	2.3	1.5
Smoking (never, past, < 15, 15–29 and ≥ 30 cigarettes/day)	23.7	6.9
Alcohol intake (never, past, < 25, 25–49 and ≥ 50 g/day)	7.9	3.2
BMI (< 22.5, 22.5–24.9, 25.0–27.4 and ≥ 27.5 kg/m^2^)	1.2	1.6
Work-related activity (quartile of MET-hr/day)	0.9	0.6
Leisure-time activity (quartile of MET-hr/week)	0.3	1.1
Coffee (0, < 1, 1–3, 4–6 and ≥ 7 cups/day)	5.7	6.8
Tea (< 1, 1–3, 4–6, 7–9 and ≥ 10 cups/day)	0.8	0.1
Diastolic blood pressure (mmHg)^b^	0.2	0.9
Antihypertensive drug (+/−)	0.7	0.0
HbA1c (%)^b^	1.0	3.1
Antidiabetic drug (+/−)	0.0	0.3
HDL cholesterol (mg/dL)^b^	2.5	10.1
Non-HDL cholesterol (mg/dL)^b^	2.9	0.2
Cholesterol-lowering drug (+/−)	2.1	0.6

BMI, body mass index; HDL, high-density lipoprotein.

^a^Proportion of partial *R*^*2*^ for a specific covariate to the overall *R*^*2*^ in the multiple linear regression including age (year) and all of the listed covariates.

^b^Continuous variables were used.

## Discussion

The present multivariate analysis showed that smoking and alcohol consumption were independent correlates of serum bilirubin in both men and women. A notable finding was that coffee consumption was associated with lower bilirubin concentrations in both sexes. Serum bilirubin showed a positive association with HDL cholesterol and a negative one with HbA1c, and the associations were more evident in women than in men.

Smoking has consistently been related to decreased levels of serum bilirubin [6-10,12,14-16]. It is postulated that bilirubin is consumed in response to increased oxidative stress due to smoking [8,9], but the exact mechanisms remain unclear. The present findings on smoking and bilirubin add to evidence for a negative association between smoking and serum bilirubin. The lack of a dose–response relationship in women was probably ascribed to chance due to fewer heavy smokers. Alternatively, female smokers may have inhaled less as compared with male smokers.

Literature on behavioral factors other than smoking is limited. While earlier studies found no clear association between alcohol drinking and serum bilirubin in either univariate or multivariate analysis [10,11,14], recent large studies suggested higher concentrations of serum bilirubin among male alcohol drinkers in Korea [12] and male and female in the United Kingdom [7]. The latter findings were also based on the univariate analysis alone [7,12]. In the present population, alcohol consumption was strongly associated with elevated concentrations of serum bilirubin in both men and women. It should be noted that a positive association between alcohol use and bilirubin among women was accentuated in the multivariate analysis while the association did not differ in the age-adjusted and multivariate-adjusted means in men. Alcohol consumption is protective against atherosclerosis, partly by increasing HDL cholesterol [30]. Subjects with known atherosclerotic diseases were excluded in the present analysis, but serum bilirubin may have reflected the extent of atherosclerotic lesions although statistical adjustment was made for important risk factors of atherosclerosis. It may be argued that deterioration of liver function underlies the positive association between alcohol and bilirubin. In fact, serum AST and ALT were positively associated with serum bilirubin in men, but not in women. However, the positive association between alcohol and bilirubin in men was not materially attenuated after adjustment for AST or ALT. Thus the alcohol-bilirubin association does not seem to be mediated by liver function deterioration.

Coffee and tea are the major sources of polyphenol intake in Japan [31]. Coffee is abundant with antioxidant polyphenols such as chlorogenic and caffeic acids while catechins are an antioxidant polyphenol in tea. Previously, coffee consumption was shown to be negatively associated with serum bilirubin as well as with serum activities of liver enzymes in a cross-sectional study in Italy [32]. Coffee consumption has consistently been associated with lower levels of ALT and AST [33], as observed in the present study population as well [34]. However, the effect of coffee on serum bilirubin seems to be mechanistically different from that on liver enzymes. As reported in the Korean study [12], serum bilirubin was minimally correlated or almost uncorrelated with ALT or AST; Spearman correlation coefficients were 0.06 for ALT and 0.11 for AST in men and −0.01 for ALT and 0.03 for AST in women. Bilirubin is produced by heme oxygenase, especially by heme oxygenase 1 induced in response to oxidative stress and other stimuli, and is excreted into bile via conjugation by uridine 5'-diphospho-glucuronosyltransferase (UGT) 1A1 [2]. Coffee compounds may affect the enzymes involved in the production or conjugation of bilirubin. Interestingly, *in vitro* and *in vivo* experiments showed that coffee infusion enhanced the expression of the *UGT1A* genes including the *UGT1A1* gene, independent of caffeine content [35]. However, such an effect of coffee on the *UGT1A1* gene was not observed in a different experimental study [36]. It deserves further research whether the negative association between coffee consumption and serum bilirubin is due to enhanced glucuronidation.

BMI was shown to be lower stepwise with higher bilirubin concentrations in women, but not in men, in some previous studies [10,12], whereas BMI was not correlated with serum bilirubin in either men or women in a national survey in the United States [6]. In a large study in the United Kingdom [7], a decrease in serum bilirubin seemed to be evident in men and women with overt obesity. In the present study, bilirubin concentrations were decreased only in women with the highest BMI (≥ 27.5 kg/m^2^). The findings are thus partly compatible with the previous observation [7,10,12].

In the Bogalusa Heart Study [13], with adjustment for sex and race, HDL cholesterol was positively correlated with serum bilirubin while non-HDL cholesterol was negatively correlated with serum bilirubin. HDL cholesterol was also shown to be positively correlated with serum bilirubin independent of smoking and physical activity in middle-aged British men [14], but total cholesterol was not an independent correlate of bilirubin among them. Neither apolipoprotein A1 nor apolipoprotein B was correlated with serum bilirubin in another multivariate analysis [15]. A positive association between HDL cholesterol and bilirubin was reported in other studies based on univariate or age-adjusted analysis [11,12], but not consistently [10]. In these studies [10-12], total cholesterol was negatively correlated with serum bilirubin except for women in one study [12]. The present study showed an independent positive association between HDL cholesterol and serum bilirubin. HDL cholesterol seemed to be more important as correlate of serum bilirubin in women than in men, but we have no clear explanation for the gender-difference in the association. As reported previously [21], HbA1c was negatively correlated with serum bilirubin, but the contribution to the variation of serum bilirubin was much smaller than HDL cholesterol.

As shown previously [24-26], hours of fasting and time of blood sampling affected bilirubin concentrations to a non-negligible extent. Importantly, however, these factors did not confound the associations between behavioral and clinical factors and serum bilirubin.

Among advantages of the present study were a large size of the study, and multivariate analysis on important correlates. Several weaknesses should be noted, however. Information on behavioral factors except BMI was based on self-report. Such information is always inaccurate to some extent, but inaccuracy was not substantial to mask the associations with alcohol and coffee consumption. Causal inference is difficult for the cross-sectional associations, particularly with laboratory measurements. Comorbid conditions may affect serum bilirubin concentrations, lifestyle factors and laboratory measurements. Thus we excluded individuals who had life-limiting morbid conditions such as atherosclerotic diseases, cancer and liver disease. Hemoglobin level was not measured in the present study.

## Conclusion

Smoking, alcohol use and coffee consumption were found to be important behavioral correlates in Japanese men and women. Serum HDL cholesterol was positively associated with serum bilirubin, being an important contributor in women.

## Abbreviations

DM: Diabetes mellitus; HDL: High-density lipoprotein; AST: Aspartate aminotransferase; ALT: Alanine aminotransferase; MET: Metabolic equivalent; BMI: Body mass index; CI: Confidence interval.

## Competing interest

The authors declare that they have no competing interests.

## Authors’ contributions

MT, AH, and SB were in charge of the development of the concept, statistical analysis and interpretation of the results, and preparing the first draft of the manuscript. MM, MA, HK and KO contributed to acquisition and compilation of the data and revision of the manuscript. RT and SK were in charge of the design and implementation of the survey and contributed to revision of the manuscript. All authors read and approved the final manuscript.

## Pre-publication history

The pre-publication history for this paper can be accessed here:

http://www.biomedcentral.com/1472-6823/13/39/prepub

## References

[B1] Vítek L Schwertner HA The heme catabolic pathway and its protective effects on oxidative stress-mediated diseases Adv Clin Chem 2007 43 1 57 17249379 10.1016/s0065-2423(06)43001-8

[B2] Schwertner HA Vítek L Gilbert syndrome, UGT1A1*28 allele, and cardiovascular disease risk: possible protective effects and therapeutic applications of bilirubin Atherosclerosis 2008 198 1 11 10.1016/j.atherosclerosis.2008.01.001 18343383

[B3] Abraham NG Kappas A Pharmacological and clinical aspects of hemo oxygenase Pharmacol Rev 2008 60 79 127 10.1124/pr.107.07104 18323402

[B4] Ohnaka K Kono S Bilirubin, cardiovascular diseases and cancer: epidemiological perspectives Expert Rev Endocrinol Metab 2010 5 891 904 10.1586/eem.10.65 30780834

[B5] Takayanagi R Inoguchi T Ohnaka K Clinical and experimental evidence for oxidative stress as an exacerbating factor of diabetes mellitus J Clin Biochem Nutr 2011 48 72 77 21297916 10.3164/jcbn.11-014FR PMC3022068

[B6] Zucker SD Horn PS Sherman KE Serum bilirubin levels in the U.S. population: gender effect and inverse correlation with colorectal cancer Hepatology 2004 40 827 835 15382174 10.1002/hep.20407

[B7] Horsfall LJ Rait G Walters K Swallow DM Pereira SP Nazareth I Petersen I Serum bilirubin and risk of respiratory disease and death JAMA 2011 305 691 697 10.1001/jama.2011.124 21325185

[B8] Van Hoydonck PG Temme EH Schouten EG Serum bilirubin concentration in a Belgian population: the association with smoking status and type of cigarettes Int J Epidemiol 2001 30 1465 1472 10.1093/ije/30.6.1465 11821365

[B9] Schwertner HA Association of smoking and low serum bilirubin antioxidant concentrations Atherosclerosis 1998 136 383 387 10.1016/S0021-9150(97)00232-3 9543110

[B10] Temme EH Zhang J Schouten EG Kesteloot H Serum bilirubin and 10-year mortality risk in a Belgian population Cancer Causes Control 2001 12 887 894 10.1023/A:1013794407325 11808707

[B11] Troughton JA Woodside JV Young IS Arveiler D Amouyel P Ferrières J Ducimetière P Patterson CC Kee F Yarnell JW Evans A Bilirubin and coronary heart disease risk in the Prospective Epidemiological Study of Myocardial Infarction (PRIME) Eur J Cardiovasc Prev Rehabil 2007 14 79 84 10.1097/01.hjr.0000230097.81202.9f 17301631

[B12] Kimm H Yun JE Jo J Jee SH Low serum bilirubin level as an independent predictor of stroke incidence: a prospective study in Korean men and women Stroke 2009 40 3422 3427 10.1161/STROKEAHA.109.560649 19713538

[B13] Bhuiyan AR Srinivasan SR Chen W Sultana A Berenson GS Association of serum bilirubin with pulsatile arterial function in asymptomatic young adults: the Bogalusa Heart Study Metabolism 2008 57 612 616 10.1016/j.metabol.2007.12.003 18442622

[B14] Breimer LH Wannamethee G Ebrahim S Shaper AG Serum bilirubin and risk of ischemic heart disease in middle-aged British men Clin Chem 1995 41 1504 1508 7586525

[B15] Ekblom K Marklund SL Jansson JH Osterman P Hallmans G Weinehall L Hultdin J Plasma bilirubin and UGT1A1*28 are not protective factors against first-time myocardial infarction in a prospective, nested case-referent setting Circ Cardiovasc Genet 2010 3 340 347 10.1161/CIRCGENETICS.109.861773 20562445

[B16] Vítek L Novotný L Sperl M Holaj R Spácil J The inverse association of elevated serum bilirubin levels with subclinical carotid atherosclerosis Cerebrovasc Dis 2006 21 408 414 10.1159/000091966 16534198

[B17] Wallner M Marculescu R Doberer D Wolzt M Wagner O Vítek L Bulmer AC Wagner KH Protection from age-related increase in lipid biomarkers and inflammation contributes to cardiovascular protection in Gilbert's syndrome Clin Sci 2013 125 257 264 10.1042/CS20120661 23566065

[B18] Ranheim T Halvorsen B Coffee consumption and human health–beneficial or detrimental?–Mechanisms for effects of coffee consumption on different risk factors for cardiovascular disease and type 2 diabetes mellitus Mol Nutr Food Res 2005 49 274 284 10.1002/mnfr.200400109 15704241

[B19] Higdon JV Frei B Coffee and health: a review of recent human research Crit Rev Food Sci Nutr 2006 46 101 123 10.1080/10408390500400009 16507475

[B20] Pham NM Yoshida D Morita M Yin G Toyomura K Ohnaka K Takayanagi R Kono S The relation of coffee consumption to serum uric Acid in Japanese men and women aged 49–76 years J Nutr Metab 2010 pub ahead of print 27 Jul 2010. doi: 10.1155/2010/930757 10.1155/2010/930757 PMC2925214 20798877

[B21] Ohnaka K Kono S Inoguchi T Yin G Morita M Adachi M Kawate H Takayanagi R Inverse associations of serum bilirubin with high sensitivity C-reactive protein, glycated hemoglobin, and prevalence of type 2 diabetes in middle-aged and elderly Japanese men and women Diabetes Res Pract 2010 88 103 110 10.1016/j.diabres.2009.12.022 20083320

[B22] Tokuda K Tanimoto K A new method of measuring bilirubin in serum by vanadic acid (Japanese) Rinsho Kagaku 1993 22 116 122

[B23] Hirata M Takanashi N Oka M Tsukada Y Application of unsensitixed soap-free latex to a new assay principle for HbA1c and its evaluation (Japanese) Igaku-to-Yakugaku 1995 34 125 136

[B24] Barrett PV Hyperbilirubinemia of fasting JAMA 1971 217 1349 1353 10.1001/jama.1971.03190100033006 5109641

[B25] Fevery J Fasting hyperbilirubinemia: unraveling the mechanism involved Gastroenterology 1997 113 1798 1800 9352889

[B26] Pocock SJ Ashby D Shaper AG Walker M Broughton PM Diurnal variations in serum biochemical and haematological measurements J Clin Pathol 1989 42 172 179 10.1136/jcp.42.2.172 2921359 PMC1141821

[B27] Broughton PM Holder R Ashby D Long-term trends in biochemical data obtained from two population surveys Ann Clin Biochem 1986 23 474 486 3767276 10.1177/000456328602300416

[B28] Kuzuya T Nakagawa S Satoh J Kanazawa Y Iwamoto Y Kobayashi M Nanjo K Sasaki A Seino Y Ito C Shima K Nonaka K Kadowaki T Report of the Committee on the classification and diagnostic criteria of diabetes mellitus Diabetes Res Clin Pract 2003 55 65 85 11755481 10.1016/s0168-8227(01)00365-5

[B29] Shimano H Arai H Harada-Shiba M Ueshima H Ohota T Yamashita S Gotoda T Kiyohara Y Hayashi T Kobayashi J Shimamoto K Bujo H Ishibashi S Shirai K Oikawa S Saito Y Yamada N Proposed guidelines for hypertriglyceridemia in Japan with non-HDL cholesterol as the second target Journal Atheroscler Thromb 2008 15 116 121 10.5551/jat.E560 18603817

[B30] Kannel WB Ellison RC Alcohol and coronary heart disease: the evidence for a protective effect Clin Chim Acta 1996 15 59 76 8814971 10.1016/0009-8981(96)06227-4

[B31] Fukushima Y Ohie T Yonekawa Y Yonemoto K Aizawa H Mori Y Watanabe M Takeuchi M Hasegawa M Taguchi C Kondo K Coffee and green tea as a large source of antioxidant polyphenols in the Japanese population J Agric Food Chem 2009 57 1253 1259 10.1021/jf802418j 19187022

[B32] Casiglia E Spolaore P Ginocchio G Ambrosio GB Unexpected effects of coffee consumption on liver enzymes Eur J Epidemiol 1993 9 293 297 10.1007/BF00146266 8104822

[B33] Cadden IS Partovi N Yoshida EM Possible beneficial effects of coffee on liver disease and function Aliment Pharmacol Ther 2007 26 1 8 17555416 10.1111/j.1365-2036.2007.03319.x

[B34] Ikeda M Maki T Yin G Kawate H Adachi M Ohnaka K Takayanagi R Kono S Relation of coffee consumption and serum liver enzymes in Japanese men and women with reference to effect modification of alcohol use and body mass index Scand J Clin Lab Invest 2010 70 171 179 10.3109/00365511003650165 20205615

[B35] Kalthoff S Ehmer U Freiberg N Manns MP Strassburg CP Coffee induces expression of glucuronosyltransferases by the aryl hydrocarbon receptor and Nrf2 in liver and stomach Gastroenterology 2010 139 1699 1710 10.1053/j.gastro.2010.06.048 20600030

[B36] Okamura S Suzuki K Yanase M Koizumi M Tamura HO The effects of coffee on conjugation reactions in human colon carcinoma cells Biol Pharm Bull 2005 28 271 274 10.1248/bpb.28.271 15684482

